# Inequities in access to health care in different health systems: a study in municipalities of central Colombia and north-eastern Brazil

**DOI:** 10.1186/1475-9276-13-10

**Published:** 2014-01-31

**Authors:** Irene Garcia-Subirats, Ingrid Vargas, Amparo Susana Mogollón-Pérez, Pierre De Paepe, Maria Rejane Ferreira da Silva, Jean Pierre Unger, Carme Borrell, Maria Luisa Vázquez

**Affiliations:** 1 Health Policy and Health Services Research Group, Health Policy Research Unit, Consortium for Health Care and Social Services of Catalonia, Avenida Tibidabo, 21, Barcelona 08022, Spain; 2 Ph. D. Programme in Biomedicine, Department of Experimental and Health Sciences, Universitat Pompeu Fabra, Barcelona, Spain; 3 Escuela de Medicina y Ciencias de la Salud, Universidad del Rosario, Calle 14, Número 6-25, Bogotá, Colombia; 4 The Prince Leopold Institute of Tropical Medicine, Nationalestraat 15, Antwerpen, Belgium; 5 FIOCRUZ/PE, Brazil, Universidade de Pernambuco, Av. Agamenon Magalhães, S/N, Recife, Brazil; 6 Agència de Salut Pública de Barcelona, Plaça Lesseps 1, Barcelona 08023, Spain; 7 Universitat Pompeu Fabra, Barcelona, Spain; 8 Ciber of Epidemiology and Public Health (CIBERESP), Madrid, Spain

**Keywords:** Access to health care, Inequities, Primary health care, Secondary care, Emergency care, Preventive health services, Colombia, Brazil

## Abstract

**Introduction:**

Health system reforms are undertaken with the aim of improving equity of access to health care. Their impact is generally analyzed based on health care utilization, without distinguishing between levels of care. This study aims to analyze inequities in access to the continuum of care in municipalities of Brazil and Colombia.

**Methods:**

A cross-sectional study was conducted based on a survey of a multistage probability sample of people who had had at least one health problem in the prior three months (2,163 in Colombia and 2,167 in Brazil). The outcome variables were dichotomous variables on the utilization of curative and preventive services. The main independent variables were income, being the holder of a private health plan and, in Colombia, type of insurance scheme of the General System of Social Security in Health (SGSSS). For each country, the prevalence of the outcome variables was calculated overall and stratified by levels of per capita income, SGSSS insurance schemes and private health plan. Prevalence ratios were computed by means of Poisson regression models with robust variance, controlling for health care need.

**Results:**

There are inequities in favor of individuals of a higher socioeconomic status: in Colombia, in the three different care levels (primary, outpatient secondary and emergency care) and preventive activities; and in Brazil, in the use of outpatient secondary care services and preventive activities, whilst lower-income individuals make greater use of the primary care services. In both countries, inequity in the use of outpatient secondary care is more pronounced than in the other care levels. Income in both countries, insurance scheme enrollment in Colombia and holding a private health plan in Brazil all contribute to the presence of inequities in utilization.

**Conclusions:**

Twenty years after the introduction of reforms implemented to improve equity in access to health care, inequities, defined in terms of unequal use for equal need, are still present in both countries. The design of the health systems appears to determine access to the health services: two insurance schemes in Colombia with different benefits packages and a segmented system in Brazil, with a significant private component.

## Introduction

In an egalitarian approach, based on the notion of social justice, equity is defined either as equal treatment for equal need (horizontal equity) or as different treatment for different needs (vertical equity) [1]. In health services research [2-7] access is usually analyzed based on the notion of horizontal equity, which would be considered to exist when access is dependent on need and not on other socioeconomic or demographic factors [8].

Colombia and Brazil are the most populous countries of Latin America. With a Gross Domestic Product (GDP) per capita of $7,752 and $11,340 (US dollars) respectively in 2012 [9,10], they are both classified as middle income countries [11]. Despite the gains in recent years in terms of poverty reduction and improvements in the Gini index [12], they still have the highest Gini coefficients in Latin America: in 2009, 0.57 [9] and 0.55 respectively [10], indicating considerable inequalities in income distribution. The illiteracy rate in Colombia (6.4%) is lower than the Latin American average (7.8%) and that of Brazil (9.6%) [13]. Public health expenditure as a percentage of the GDP was 4.6% in Colombia and 4.1% [9] in Brazil in 2011 [10]. They have similar life expectancy at birth (74.0 in Colombia and 73.9 in Brazil) which is close to the Latin American average [13], while the infant mortality rate is slightly higher in Colombia (18.4 per 1000 live births) than in Brazil (15.3 per 1000 live births) or Latin America in general (16.3 per 1000 live births) [13].

Both countries reformed their health systems more than two decades ago with the common objective (among others) of improving equity of access, but they opted for different models. Colombia introduced the General System of Social Security in Health (*Sistema General de Seguridad Social en Salud* or *SGSSS* in Spanish), based on managed competition and made up of two insurance schemes: the contributory scheme, for formal sector employees and those able to pay, and the subsidized scheme for the low income population. Those that do not manage to enroll in either of the two schemes remain uninsured. Health insurance is managed by healthcare insurers (*Empresas Promotoras de Salud* or EPS in Spanish) for both the contributory scheme (EPS-C) and the subsidized scheme (EPS-S). Competition was introduced between insurers for the enrollment of the population and they receive a capitation payment to cover different benefits packages in each scheme: the Obligatory Health Plan (*Plan Obligatorio de Salud* or POS in Spanish) for the contributory scheme and the Obligatory Health Plan - Subsidized (POS-S) for the subsidized scheme [14], with fewer services than the POS. The insurers are responsible for organizing their own network of health providers for their enrollees. The providers are different for the two schemes.

Competition for contracts with the insurers was also introduced among public and private healthcare providers (*Instituciones Prestadoras de Salud* or IPS in Spanish). The uninsured population, 12.1% [15], receives care in public healthcare service networks which are organized by regional and local health authorities [16].

In Brazil, the health sector is made up of two subsystems: on one side, the Unified Health System (*Sistema Único de Saúde* or *SUS* in Portuguese), conceived as a tax-funded national health system, decentralized according to the political structure of the country (federation, states and municipalities) and free at point of delivery [17], with care provided by public or contracted private providers; and on the other side, the private system (supplementary system), to which the population gains access via direct payment or private insurance schemes, which have their own network provider.

In both countries, care is organized by levels of complexity, with primary care as the entry point and care coordinator for the patient and the secondary level in a supporting role [16,18]. In the private healthcare subsystem, outpatient secondary care services can be accessed directly (via a private health plan or out-of-pocket payment).

The effects of these reforms on access in Brazil and Colombia have been analyzed indirectly through studying the design of the health system [19,20], changes in coverage in the family health program in Brazil [21], or enrollment with an insurer in Colombia [22], and in a more direct way through looking at changes in utilization and equity in utilization before and after the reforms [23,24]. There are also studies available for both countries analyzing equity in the utilization of the health services, mostly based on national surveys of the general population: the Quality of Life Survey (*Encuesta de Calidad de Vida, ECV)* and the Demography and Health Survey (*Encuesta de Demografía y Salud*, EDS) in Colombia, and the National Household Sample Survey *(Pesquisa Nacional de Amostra por Domicilios*, PNAD) in Brazil. These studies indicate that, irrespective of need, individuals of a higher socioeconomic status – higher income [5,7,24-27], higher level of education [23,25,28,29] or more favorable working conditions (stable employment) [30] – make greater use of the health services. In Brazil, people who have private medical insurance are shown to be more likely to use the health services [31]. These analyses, however, have certain limitations. In both countries, the analysis of equity in access focuses on the utilization of services in general [5,6,26,27], without distinguishing between care levels, despite the international evidence showing that inequities vary between primary and secondary care [32-34], with the inequities being more pronounced in the latter. Only one study has been found for Colombia which differentiates utilization according to care levels [7] and there are none for Brazil. Likewise, equity in access to preventive services [28,35,36] is a little explored subject matter. In Colombia, analyses also tend to focus on the differences between those covered by the SGSSS –either in general [24] or in the subsidized scheme [37,38]- and the uninsured, whilst studies comparing the use of services in the different insurance schemes are rare and give conflicting results [27,28,39].

In both countries the studies tend to analyze inequities between large geographical regions (Departments or States) [40,41], but not between smaller areas within these, although there are signs of inequities in access within regions, for example between the different localities in the city of Bogotá [42], a region with one of the highest percentages of health services utilization in Colombia [15]. In Brazil, the studies which analyze smaller areas mainly focus on the south of the country [43-46], and there are practically none for the northeast, despite this region having lower utilization rates than the national average and greater inequity in the use of the health services [40].

Lastly, equity of access in the health systems of Colombia and Brazil has been analyzed in the context of Latin American-wide studies which compare either the design of different health systems [1,47-49] or equity in utilization [4,41,50]. These are generally based on national surveys which were not designed for this purpose, so differences in the questions asked make them difficult to compare [4,41]. The only comparative study between the two countries analyzes inequities in health [51], but not in access to services.

Identifying the care levels in which there is inequity in utilization (and to what degree) in different health systems may contribute to the design of policies aimed at reducing this problem. The purpose of this study is to conduct a comparative analysis of equity in access to the health services distinguishing between different care levels (primary, outpatient secondary, emergency and preventive care) in two areas of Colombia and Brazil, focusing particularly on the role of insurance scheme enrollment in Colombia and the possession of a private health plan in Brazil.

## Methods

### Design and study area

A cross-sectional study was conducted by means of a population survey in central Colombia and the northeast of Brazil. The study areas were two municipalities in each country: in Colombia, Kennedy (a district of Bogotá) and Soacha; and in Brazil, two micro-regions (3.2 and 3.3) of District 3 in Recife, Pernambuco’s capital, and Caruaru, in the interior of Pernambuco state. These four areas are the areas of the Equity-LA project ( http://www2.equity-la.eu/), the broader Project in which this study is framed [52]. The areas were selected for being densely populated urban spaces with a high proportion of the population belonging to the low or medium-low socioeconomic strata and with varying geographical access to specialist care. Kennedy has approximately 1,000,000 residents and the other three study areas have about 300,000 residents. Kennedy and the two micro-regions of District 3 in Recife share the characteristic of being some of the more deprived areas in their respective cities.

### Study population and sample

The study population was made up of residents in the study areas who had had at least one health problem or had used to the health services in the three months prior to the survey.

The sample size was calculated for each study area based on the population size and an estimated proportion of 50% (maximum uncertainty principle); degree of confidence: 90% (alpha error of 0.1); precision: 2.5. The sample size was 2,163 in Colombia (1,083 in Kennedy, 1,080 in Soacha) and 2,155 in Brazil (1,076 in district 3 of Recife, 1,079 in Caruaru).

In both countries, multistage probability sampling was conducted. In the first stage, census tracts were randomly selected (in Soacha, from all six *comunas* – i.e. districts) with replacement. In the second stage, households were systematically selected. The sample range was calculated according to sample size and number of households in each neighborhood; the initial home was randomly selected. The household was considered the primary sampling unit to avoid the effect of associated samples in individuals belonging to a family.

### Questionnaire

A questionnaire was designed to analyze access to health care. Based on the Behavioral Model of Health Services Use [53] and previous qualitative research [54-58], dimensions and variables were identified. In addition, systematic reviews were conducted of studies on access and equity of access to health care and of available tools in order to identify additional variables. The literature search was carried out using the most relevant electronic databases (Pubmed, CINAHL, Social Science Citation Index, PsycInfo, Lilacs, IBECS, The Cochrane Library, System for Information on Grey Literature in Europe, Information system of the WHO Library, Panamerican Health Organization Library) to minimize the likelihood of excluding relevant studies. The search strategy included the combination of descriptors and keywords relating to equity in access to health care, delivery of health care and measurement instruments, utilizing the Boolean operator ‘AND’. Given the large number of studies identified, only studies from Colombia, Brazil, United States and Spain were included. The U.S. and Spain were included because their health systems are comparable to the Colombian and Brazilian system respectively. A total of 41 different articles were identified up to 2010. Based on these results, the first version of the questionnaire was built and validated by means of discussions with experts, after which it was adapted to both context and languages. A pre-test and a pilot test were conducted in each country to evaluate the rhythm of the interview, interviewer burden and comprehensibility. Both the rhythm of the interview and the interviewer burden proved adequate. With respect to comprehensibility, some questions were removed and others modified because the terms used were unfamiliar and people had problems understanding them. Finally, since many changes had been made, another pilot was deemed necessary, in which the questionnaire was finally considered to be adequate. The final questionnaire is divided into nine sections. The first collects information on perceived health needs and related behavior in the three months prior to the survey, in other words, whether or not the individual had used the health services and the level of care at which they had been attended. The next four sections refer to their most recent experience - within the three months prior to the survey - using the different levels of health care (primary, outpatient secondary, emergency, and inpatient care) of the SGSSS and SUS services. The last three sections include a Likert scale to measure care continuity, knowledge of the healthcare system and sociodemographic data. The Colombian questionnaire has an additional section related to insurance enrollment.

### Data collection

Data were collected from February to June 2011 by means of face-to-face interviews conducted by specifically trained interviewers in both countries.

Strategies to ensure the quality and consistency of data included close supervision of interviewers in the field, the review of all questionnaires and 20% of re-interviews (selected randomly). Inconsistencies during data entry were controlled through the double-entry method.

### Ethical considerations

Ethics approval was obtained from the ethics committees in the participating countries: the National Committee of Research Ethics in Brazil; the Research Ethics Committee of the Health Sciences School of Universidad del Rosario in Colombia; the Institutional Review Board of the Institute of Tropical Medicine in Belgium; and the Clinical Research Ethics Committee of Parc de Salut Mar in Spain. All interviewees participated on a voluntary basis, after signing an informed consent. The right to refuse to participate or withdraw from the survey, anonymity, confidentiality and protection of data were all guaranteed.

### Variables

The outcome variables are three dichotomous variables on utilization of healthcare services in the three months prior to the survey: a) consultation of a general practitioner or pediatrician, b) consultation of a specialist, c) consultation of emergency care services; and three variables on the use of preventive services by adults in the last year: d) glycemic control, e) caries prevention and f) mammography in women.

Need for care was measured by self-rated health (dichotomous: i) good - very good and good, and ii) poor - fair, poor and very poor) and having at least one chronic condition [59]. The main explanatory variables were per capita income and holding a private health plan in both countries as a proxy of socio-economic status, and type of SGSSS insurance scheme in Colombia. Per capita income (less than half minimum wage (MW), ½ - 1 MW, 2 or more MWs) was estimated by dividing the household income by the family size. Sociodemographic variables - sex, age (0–17, 18–40, 41–65, 66 or over) acolor/color- were used to adjust the models.

### Data analysis

A univariate analysis was performed to describe the distribution of the outcome and explanatory variables for each country (Table 1). To establish the relative association between each healthcare utilization variable and socioeconomic status, prevalence ratios (PR) and their corresponding 95% confidence intervals were computed by means of Poisson regression models with robust variance (Tables 2 and 3). PR was considered better suited to our study than other measures of inequality such as the Relative Index of Inequality (RII) because the socio-economic variables included in the models are not strictly hierarchical [60]. Absolute differences in utilization prevalence at both extremes of the three socioeconomic variables were also calculated. Both absolute and relative measures were used to assess socioeconomic inequalities in the utilization of healthcare services.

**Table 1:** Sociodemographic characteristics and perceived health care need of the study sample, in the study areas of Colombia and Brazil (2011)

	**Colombia (n = 2,163)**		**Brazil (n = 2,155)**	
	**n**	**%**	**n**	**%**
**Sex**				
Male	691	31.9	626	29.0
Female	1,472	68.1	1,529	71.0
**Age**				
0-17	300	13.9	483	22.4
18-40	667	30.8	497	23.1
41-65	909	42.0	766	35.6
>65	287	13.3	409	19.0
**Level of education**				
None	344	16.0	623	29.5
Primary	804	37.4	583	27.6
Secondary	821	38.2	798	37.8
University	180	8.4	106	5.0
**Per capita income**				
< ½ MW	951	44.0	1,121	52.0
½ - 1 MW	775	35.8	720	33.4
> 1 MW	437	20.2	314	14.6
**Private health plan**				
Yes	42	2.0	434	20.1
No	2,024	98.0	1,721	79.9
**SGSSS scheme**				
Contributory	1,144	56.0		
Subsidized	574	28.0		
Special	97	4.8		
Uninsured	231	11.3		
**Self-rated health status**				
Good	1,346	62.3	962	44.7
Poor	816	37.7	1,192	55.3
**Chronic condition**				
Yes (at least one)	650	30.0	903	41.9
No	1,513	70.0	1,252	58.1

MW, minimum wage; SGSSS, General System of Social Security in Health.

**Table 2:** **Prevalence, prevalence difference and prevalence ratios (95**% **confidence intervals) of health services utilization in the last three months by per capita income, SGSSS insurance scheme and private health plan, in the study areas of Colombia and Brazil (2011)**

	**Colombia**				**Brazil**			
	**n**	**Prev.**	**PD**	**PR (CI 95%)**^**a**^	**n**	**Prev.**	**PD**	**PR (CI 95%)**^**a**^
**Primary care**								
*Per capita income*								
< 1/2 MW	473	49.8		1	550	49.1		1
1/2 - 1 MW	409	52.8	3.0	1.04 (0.94 - 1.14)	310	43.1	-6.0	**0.89 (0.80 - 0.99)**
> 1 MW	246	56.4	6.6	1.01 (0.91 - 1.13)	98	31.2	-17.9	**0.71 (0.59 - 0.85)**
*SGSSS scheme*								
Contributory + special	718	58.0		1				
Subsidized	298	52.1	-5.9	**0.90 (0.81 - 0.99)**				
Uninsured	57	24.7	-33.3	**0.46 (0.37 - 0.58)**				
*Private Health Plan*								
No	1,041	51.5		1	800	46.5		1
Yes	27	64.3	12.8	1.13 (0.90 - 1.41)	158	36.4	-10.1	0.91 (0.79 - 1.05)
**Outpatient secondary care**								
*Per capita income*								
< 1/2 MW	160	16.9		1	228	20.3		**1**
1/2 - 1 MW	150	19.4	2.5	1.02 (0.83 - 1.26)	200	27.8	7.5	**1.26 (1.07 - 1.48)**
> 1 MW	118	27.1	10.2	**1.26 (1.02 - 1.56)**	96	30.6	10.3	**1.42 (1.14 - 1.77)**
*SGSSS scheme*								
Contributory + special	300	24.2		1				
Subsidized	93	16.3	-7.9	**0.72 (0.57 - 0.90)**				
Uninsured	17	7.4	-16.8	**0.40 (0.25 - 0.64)**				
*Private Health Plan*								
No	393	19.5		1	391	22.7		1
Yes	16	38.1	18.6	**1.74 (1.14 - 2.66)**	133	30.7	8.0	**1.40 (1.18 - 1.67)**
**Emergency care**								
*Per capita income*								
< 1/2 MW	192	20.2		1	390	34.8		1
1/2 - 1 MW	141	18.2	-2.0	1.14 (0.92 - 1.41)	240	33.3	-1.5	1.09 (0.95 - 1.25)
> 1 MW	70	16.1	-4.1	0.98 (0.74 - 1.29)	74	23.6	-11.2	0.88 (0.70 - 1.11)
*SGSSS scheme*								
Contributory + special	230	18.6		1				
Subsidized	123	21.5	2.9	0.94 (0.76 - 1.17)				
Uninsured	33	14.3	-4.3	**0.63 (0.45 -0.88)**				
*Private Health Plan*								
No	371	18.4		1	579	33.6		1
Yes	12	28.6	10.2	**1.75 (1.06 - 2.86)**	125	28.8	-4.8	0.93 (0.78 - 1.11)

Prev, Prevalence; PD, Prevalence Difference; PR, Prevalence ratio; CI, Confidence Interval; MW, minimum wage; SGSSS, General System of Social Security in Health.

^a^Prevalence Ratio and 95% confidence interval adjusted for sex, age, race/color, chronic condition, self-rated health status.

Statistically significant results are shown in bold.

**Table 3:** **Prevalence, prevalence difference and prevalence ratios (95**% **confidence intervals) of preventive care activities in the last year by per capita income, SGSSS insurance scheme and private health plan, in the study areas of Colombia and Brazil (2011)**

	**Colombia**				**Brazil**			
	**n**	**Prev**	**PD**	**PR (CI 95%)**^**a**^	**n**	**Prev**	**PD**	**PR (CI 95%)**^**a**^
**Glycemic control (adults)**								
*Per capita income*								
< 1/2 MW	441	49.8		1	427	52.6		1
1/2 - 1 MW	275	47.5	-2.3	0.97 (0.87 - 1.08)	346	58.8	6.2	**1.11 (1.01 - 1.21)**
> 1 MW	230	57.6	7.8	1.09 (0.98 - 1.22)	160	58.8	6.2	1.11 (0.98 - 1.26)
*SGSSS scheme*								
Contributory + special	626	56.6		1				
Subsidized	223	47.8	-8.8	**0.83 (0.75 - 0.93)**				
Uninsured	54	28.3	-28.3	**0.59 (0.47 - 0.75)**				
*Private Health Plan*								
No	890	50.9		1	726	53.6		1
Yes	19	54.3	3.4	0.98 (0.73 - 1.33)	207	65.1	11.5	**1.26 (1.14 - 1.40)**
**Caries prevention (adults)**								
*Per capita income*								
< 1/2 MW	308	34.8		1	123	15.2		1
1/2 - 1 MW	232	40.1	5.3	1.13 (0.98 - 1.30)	129	21.9	6.7	**1.34 (1.07 - 1.67)**
> 1 MW	162	40.6	5.8	1.12 (0.94 - 1.32)	81	29.8	14.6	**1.41 (1.08 - 1.84)**
*SGSSS scheme*								
Contributory + special	437	39.5		1				
Subsidized	172	36.8	-2.7	0.95 (0.81 - 1.11)				
Uninsured	59	30.9	-8.6	**0.75 (0.60 - 0.94)**				
*Private Health Plan*								
No	658	37.6		1	218	16.1		1
Yes	15	42.9	5.3	1.15 (0.80 - 1.65)	115	36.2	20.1	**1.86 (1.52 - 2.28)**
**Breast cancer prevention (women)**								
*Per capita income*								
< 1/2 MW	144	21.9		1	168	26.3		1
1/2 - 1 MW	120	30.0	8.1	**1.27 (1.04 - 1.57)**	128	27.9	1.6	1.10 (0.90 - 1.33)
> 1 MW	96	37.2	15.3	**1.37 (1.10 - 1.71)**	69	37.3	11.0	**1.47 (1.14 - 1.90)**
*SGSSS scheme*								
Contributory + special	261	34.7		1				
Subsidized	63	17.8	-16.9	**0.59 (0.46 - 0.76)**				
Uninsured	15	11.0	-23.7	**0.43 (0.27 - 0.70)**				
*Private Health Plan*								
No	344	27.8		1	276	26.5		1
Yes	9	36.0	8.2	0.99 (0.58 - 1.67)	89	36.8	10.3	**1.38 (1.11 - 1.70)**

Prev, Prevalence; PD, Prevalence Difference; PR, Prevalence ratio; CI, Confidence Interval; MW, minimum wage; SGSSS, General System of Social Security in Health.

^a^Prevalence Ratio and 95% confidence interval adjusted for sex, age, race/color, chronic condition, self-rated health status.

Statistically significant results are shown in bold.

In addition, stratified analyses were carried out: firstly, regression models were generated stratifying by income to evaluate the effect of the insurance scheme in Colombia and additional private health insurance in Brazil on health services utilization for each socioeconomic stratum (Tables 4 and 5); and secondly, regression models were made stratifying by type of insurance in both countries (contributory and subsidized in Colombia, and only SUS or SUS and private health plan in Brazil) (Table 6). Analyses were carried out with STATA statistical package version 12 [61].

**Table 4:** Prevalence, prevalence difference and prevalence ratios (95% confidence intervals) of health services utilization in the last three months and of preventive activities in the last year by SGSSS insurance scheme, according to per capita income, in the study areas of Colombia (2011)

	**< ½ MW (per capita)**				**½ - 1 MW (per capita)**				**> 1 MW (per capita)**			
	**n**	**Prev**	**PD**	**PR (CI 95%)**^**a**^	**n**	**Prev**	**PD**	**PR (CI 95%)**^**a**^	**n**	**Prev**	**PD**	**PR (CI 95%)**^**a**^
**Primary care**												
Contributory + special	261	57.9		1	243	58.0		1	214	58.0		1
Subsidized	210	51.0	-6.9	0.88 (0.78 - 1.01)	70	54.7	-3.3	0.92 (0.77 - 1.10)	18	56.3	-1.7	0.91(0.65 - 1.29)
Uninsured	37	25.3	-32.6	**0.48 (0.35 - 0.64)**	14	20.9	-37.1	**0.41 (0.26 - 0.65**)	6	33.3	-24.7	0.59 (0.31 - 1.13)
**Outpatient secondary care**												
SGSSS scheme												
Contributory + special	105	23.3		1	89	21.2		1	106	28.7		1
Subsidized	65	15.8	-7.5	**0.70 (0.53 - 0.94)**	19	14.8	-6.4	**0.59 (0.38 - 0.91**)	9	28.1	-0.6	1.16 (0.66 - 2.05)
Uninsured	12	8.2	-15.1	**0.45 (0.25 - 0.81)**	5	7.5	-13.7	**0.38 (0.16 - 0.88**)	0	0	--	--
**Emergency care**												
Contributory + special	87	19.3		1	84	20.1		1	59	16.0		1
Subsidized	91	22.1	2.8	0.92 (0.70 - 1.21)	28	21.9	1.8	1.04 (0.71 - 1.54)	4	12.5	-3.7	0.60 (0.21 - 1.70)
Uninsured	23	15.8	-3.5	0.67 (0.44 - 1.01)	7	10.5	-9.6	0.52 (0.26 - 1.05)	3	16.7	0.7	0.97 (0.37 - 2.57)
**Glycemic control (adults)**												
Contributory + special	230	58.4		1	188	50.4		1	208	61.4		1
Subsidized	152	46.9	-11.5	**0.80 (0.69 - 0.92)**	57	50.0	-0.4	0.90 (0.74 - 1.10)	14	48.3	-13.1	0.83 (0.57 - 1.23)
Uninsured	33	29.0	-29.4	**0.61 (0.45 - 0.82)**	19	31.7	-18.7	0.70 (0.48 - 1.04)	2	11.8	-49.6	0.23 (**0.06 - 0.82**)
**Caries prevention (adults)**												
Contributory + special	149	37.8		1	149	40.0		1	139	41.0		1
Subsidized	108	33.3	-4.5	0.83 (0.67 - 1.03)	51	44.7	4.7	1.13 (0.89 - 1.44)	13	44.8	3.8	1.06 (0.68 - 1.64)
Uninsured	32	28.1	-9.7	**0.70 (0.51 - 0.97)**	22	36.7	-3.3	0.84 (0.58 - 1.20)	5	29.4	-11.6	0.64 (0.30 - 1.33)
**Breast cancer prevention (women)**												
Contributory + special	88	31.3		1	86	33.6			87	40.5		1
Subsidized	32	12.8	-18.5	**0.46 (0.31 - 0.66)**	25	31.3	-2.3	0.82 (0.57 - 1.16)	6	26.1	-14.4	0.68 (0.35 - 1.34)
Uninsured	9	10.5	-20.8	**0.48 (0.26 - 0.88)**	5	12.8	-20.8	0.42 (0.18 - 1.00)	1	8.3	-32.2	0.28 (0.04 - 2.03)

Prev, Prevalence; PD, Prevalence Difference; PR, Prevalence ratio; CI, Confidence Interval; MW, minimum wage; SGSSS, General System of Social Security in Health.

^a^Prevalence Ratio and 95% confidence interval adjusted for sex, age, race/color, chronic condition, self-rated health status.

Statistically significant results are shown in bold.

**Table 5:** **Prevalence, prevalence difference and prevalence ratios (95**% **confidence intervals) of health services utilization in the last three months and of preventive activities in the last year by private health plan (PHP), according to per capita income, in the study areas of Brazil (2011)**

	**< ½ MW (per capita)**				**½ - 1 MW (per capita)**				**> 1 MW (per capita)**			
	**n**	**Prev**	**PD**	**PR (CI 95%)**^**a**^	**n**	**Prev**	**PD**	**PR (CI 95%)**^**a**^	**n**	**Prev**	**PD**	**PR (CI 95%)**^**a**^
**Primary care**												
Without PHP	506	49.3		**1**	247	45.2		1	47	32.0		1
With PHP	44	46.8	-2.5	0.99 (0.79 - 1.25)	63	36.4	-8.8	**0.79 (0.64 - 0.99)**	51	30.5	-1.5	0.95 (0.68 - 1.33)
**Outpatient secondary care**												
Without PHP	212	20.6		1	141	25.8		**1**	38	25.9		1
With PHP	16	17.0	-3.6	0.98 (0.64 - 1.52)	59	34.1	8.3	**1.57 (1.23 - 2.00)**	58	34.7	8.8	1.40 **(1.02 - 1.93)**
**Emergency care**												
Without PHP	348	33.9		1	194	35.5		1	37	25.2		1
With PHP	42	44.7	10.8	**1.35 (1.05 - 1.72)**	46	26.6	-8.9	**0.66 (0.50 - 0.86)**	37	22.2	-3.0	1.03 (0.68 - 1.55)
**Glycemic control (adults)**												
Without PHP	391	52.1		1	268	57.0		1	67	50.0		1
With PHP	36	58.1	6.0	1.16 (0.93 - 1.45)	78	66.1	9.1	**1.22 (1.06 - 1.42)**	93	67.4	17.4	**1.40 (1.15 - 1.71)**
**Caries prevention (adults)**												
Without PHP	107	14.3		1	88	18.7		1	23	17.2		1
With PHP	16	25.8	11.5	**1.80 (1.15 - 2.82)**	41	34.8	16.1	**1.58 (1.17 - 2.12)**	58	42.0	24.8	**2.42 (1.59 - 3.68)**
**Breast cancer prevention (women)**												
Without PHP	149	25.3		1	100	27.4		1	27	31.0		1
With PHP	19	38.0	12.7	**1.61 (1.09 - 2.36)**	28	29.8	2.4	**1.15 (0.81 - 1.63)**	42	42.9	11.9	**1.48 (1.02 - 2.15)**

Prev, Prevalence; PD, Prevalence Difference; PR, Prevalence ratio; CI, Confidence Interval; MW, minimum wage; PHP, Private Health Plan.

^a^Prevalence Ratio and 95% confidence interval adjusted for sex, age, race/color, chronic condition, self-rated health status.

Statistically significant results are shown in bold.

**Table 6:** **Prevalence, prevalence difference and prevalence ratios (95**% **confidence intervals) of health services utilization in the last three months and of preventive activities in the last year by per capita income according to SGSSS insurance scheme and private health plan, in the study areas of Colombia and Brazil (2011)**

	**Colombia**								**Brazil**							
	**Contributory scheme**				**Subsidized scheme**				**Without private health plan**				**With private health plan**			
	**n**	**Prev**	**PD**	**PR (CI 95%)**^**a**^	**n**	**Prev**	**PD**	**PR (CI 95%)**^**a**^	**n**	**Prev**	**PD**	**PR (CI 95%)**^**a**^	**n**	**Prev**	**PD**	**PR (CI 95%)**^**a**^
**Primary care**																
< 1/2 MW	261	57.9		1	210	51.0		1	506	49.3		1	44	46.8		1
1/2 - 1 MW	243	58.0	0.1	1.05 (0.94 - 1.17)	70	54.7	3.7	1.08 (0.90 - 1.29)	247	45.2	-4.1	0.91 (0.81 - 1.02)	63	36.4	-10.4	0.75 (0.56 - 1.02)
> 1 MW	214	58.0	0.1	1.03 (0.92 - 1.16)	18	56.3	5.3	1.07 (0.78 - 1.48)	47	32.0	-17.3	**0.69 (0.54 - 0.88)**	51	30.5	-16.3	**0.66 (0.48 - 0.93)**
**Outpatient secondary care**																
< 1/2 MW	105	23.3		1	65	15.8		1	212	20.6		1	16	17.0		1
1/2 - 1 MW	89	21.2	-2.1	1.02 (0.80 - 1.29)	19	14.8	-1.0	0.87 (0.55 - 1.39)	141	25.8	5.2	1.17 (0.98 - 1.41)	59	34.1	17.1	**1.94 (1.22 - 3.10)**
> 1 MW	106	28.7	5.4	**1.26 (1.01 - 1.58)**	9	28.1	12.3	1.72 (0.98 - 3.02)	38	25.9	5.3	1.34 (1.00 - 1.79)	58	34.7	17.7	**1.98 (1.23 - 3.20)**
**Emergency care**																
< 1/2 MW	87	19.3		1	91	22.1		1	348	33.9		1	42	44.7		1
1/2 - 1 MW	84	20.1	0.8	1.11 (0.85 - 1.45)	28	21.9	-0.2	1.19 (0.82 - 1.73)	194	35.5	1.6	**1.19 (1.04 - 1.38)**	46	26.6	-18.1	**0.63 (0.45 - 0.87)**
> 1 MW	59	16.0	-3.3	0.95 (0.70 - 1.29)	4	12.5	-9.6	0.72 (0.29 - 1.75)	37	25.2	-8.7	0.90 (0.67 - 1.22)	37	22.2	-22.5	0.70 (0.48 - 1.01)
**Glycemic control (adults)**																
< 1/2 MW	230	58.4		1	152	46.9		1	391	52.1		1	36	58.1		1
1/2 - 1 MW	188	50.4	-8.0	0.93 (0.82 - 1.05)	57	50.0	3.1	1.07 (0.87 - 1.31)	268	57.0	4.9	1.11 (1.00 - 1.23)	78	66.1	8.0	1.15 (0.90 - 1.47)
> 1 MW	208	61.4	3.0	1.11 (0.99 - 1.25)	14	48.3	1.4	1.03 (0.71 - 1.51)	67	50.0	-2.1	1.05 (0.87 - 1.26)	93	67.4	9.3	1.21 (0.95 - 1.53)
**Caries prevention (adults)**																
< 1/2 MW	149	37.8		1	108	33.3		1	107	14.3		1	16	25.8		1
1/2 - 1 MW	149	40.0	2.2	1.02 (0.85 - 1.22)	51	44.7	11.4	**1.45 (1.12 - 1.87)**	88	18.7	4.4	**1.37 (1.06 - 1.76)**	41	34.8	9.0	1.34 (0.83 - 2.17)
> 1 MW	139	41.0	3.2	1.06 (0.88 - 1.27)	13	44.8	11.5	1.45 (0.97 - 2.16)	23	17.2	2.9	1.11 (0.73 - 1.69)	58	42.0	16.2	**1.87 (1.16 - 3.01)**
**Breast cancer prevention (women)**																
< 1/2 MW	88	31.3		1	32	12.8		1	149	25.3		1	19	38.0		1
1/2 - 1 MW	86	33.6	2.3	1.13 (0.89 - 1.42)	25	31.3	18.5	**2.00 (1.28 - 3.11)**	100	27.4	2.1	1.20 (0.97 - 1.47)	28	29.8	-8.2	0.77 (0.48 - 1.23)
> 1 MW	87	40.5	9.2	**1.35 (1.07 - 1.69)**	6	26.1	13.3	1.69 (0.82 - 3.45)	27	31.0	5.7	**1.50 (1.05 - 2.14)**	42	42.9	4.9	1.10 (0.73 - 1.68)

Prev, Prevalence; PD, Prevalence Difference; PR, Prevalence ratio; CI, Confidence Interval; MW, minimum wage.

^a^Prevalence Ratio and 95% confidence interval adjusted for sex, age, race/color, chronic condition, self-rated health status.

Statistically significant results are shown in bold.

## Results

*Colombia* and *Brazil* are used here to refer to the study areas even though the results are not extrapolated at country level.

### Sample characteristics

In both samples, the majority of participants are women, and while in the Colombia sample there is a predominance of the central age groups (18 to 40 and 41 to 65 years), in that of Brazil there is a more even distribution of the sample across all age groups. With regard to SGSSS enrollment, 56.0% are enrolled in the contributory scheme, 28.0% in the subsidized scheme, and 11.3% are uninsured. The socioeconomic status of the sample is not very high in either country: most people have less than half of the minimum wage (MW) per month, but distribution is less equal in Brazil than in Colombia. The socioeconomic level in Colombia is closely related to enrollment; the proportion of people with less than half MW in the previous month is higher in the subsidized scheme and the uninsured group than in the contributory scheme: 72.0%, 63.2% and 36.4% respectively. In Colombia 2.0% and in Brazil 20.1% hold a private health plan (PHP) (Table 1). Self-rated health status is better in Colombia: 62.2% report good or very good health, compared to 44.7% in Brazil; 30.0% claim to have at least one chronic disease, as opposed to 41.9% in Brazil (Table 1).

### Use of health care services

**Figure 1:**
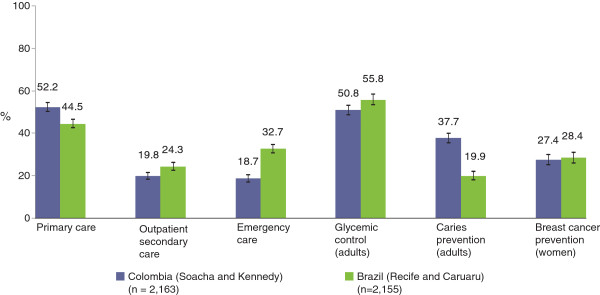
Prevalence of health services utilization in the last three months and of preventive care activities in the last year, study areas of Colombia and Brazil (2011).

In the Colombia sample more than half of the individuals used the first level of care (52.2%), a higher proportion than in that of Brazil (44.5%). However, for outpatient secondary and emergency care, utilization in Brazil was significantly higher than in Colombia (Figure 1). Preventive activities in the last year differ between countries according to type of activity: while the proportions of people who used glycemic control and mammography services were similar, significant differences were found in caries prevention in adults (37.7% in Colombia vs. 19.9% in Brazil) (Figure 1).

### Differences in socioeconomic-related inequalities in health services utilization

At primary care level, per capita income does not figure as a source of inequality in Colombia but it does in Brazil, where individuals with a higher per capita income make less use of this care level (prevalence difference with respect to the poorest is -17.9, PR = 0.71, CI 95%: 0.59-0.85). In Colombia, it is the insurance scheme which is associated with the utilization of services at this level. The utilization prevalence of individuals in the contributory scheme is 58.0%, in the subsidized scheme 52.1%, and 24.7% for the uninsured (Table 2). In outpatient secondary care, per capita income is a source of inequity in both countries: higher-income individuals are more likely to use these services. In Colombia, the PR of individuals with more than one minimum wage per capita to those with the lowest income is 1.26 (CI 95%: 1.02-1.56) and in Brazil, 1.42 (CI 95%: 1.14-1.77), which is the opposite pattern of use to that detected at primary care level. Holding a private health plan is also associated with a higher prevalence of utilization in both countries. Furthermore, in Colombia, insurance enrollment reveals the same pattern as that found in primary care. Individuals in the contributory scheme display the highest use of this care level: the PR of subsidized to contributory is 0.72 (CI 95%: 0.57-0.90) and of uninsured to contributory is 0.40 (CI 95%; 0.24-0.64) (Table 2). With regard to emergency care services, the higher the per capita income is, the lower the prevalence of utilization in both countries, although once this is adjusted for need there are no perceivable inequities associated with income and private insurance in either country. However, in Colombia individuals not insured under the SGSSS are unfavorably positioned with respect to those insured under the contributory scheme (PR: 0.63, CI 95% 0.45-0.88) (Table 2).

There are noticeable inequities in preventive activities in both countries. The absolute differences in utilization prevalence (of all three socioeconomic variables) are always in favor of those who are most socioeconomically favored. In Colombia, uninsured individuals are less likely to use the three preventive services than those of the contributory scheme. Moreover, individuals in the subsidized scheme are less likely to have glycemic controls and mammographies than individuals in the contributory scheme. The inequities are accentuated in breast cancer prevention according to per capita income: the prevalence of utilization increases as the level of income rises (21.9%, 30.0% and 37.2%). In Brazil, people with a lower income and without private insurance are less likely to use the three preventive services, and the difference in prevalence of using caries prevention services is particularly marked between those without private insurance (16.1%) and those with (36.2%) (Table 3).

### What are the effects of type of SGSSS insurance scheme and private health plan at each level of income?

This second section looks closely at the effects in each income stratum of insurance scheme in Colombia and holding a private health plan in Brazil on the utilization of healthcare services, after having observed an association between higher socioeconomic status and greater use of the health services. In Colombia, in the two lower-income strata, individuals of the contributory scheme are more likely to use the primary and outpatient secondary care services than the uninsured; and they are also more likely to use outpatient secondary care than individuals in the subsidized scheme (PR = 0.70, CI 95% 0.53-0.94 in the lower per capita income stratum and PR = 0.59, CI 95%: 0.38-0.91 in the middle stratum). Inequalities between schemes were not found in the case of emergency care (Table 4). The effect of the insurance scheme on the use of preventive services is particularly noticeable in the lowest per capita income stratum, where belonging to the subsidized scheme or being uninsured significantly reduces the probability of using glycemic control services (prevalence difference between individuals in the contributory scheme and in the subsidized scheme is -11.5, and between individuals in the contributory scheme and the uninsured is -29.4) and mammography services (prevalence difference is -18.5 for the subsidized enrollees and -20.8 for the uninsured) (Table 4).

In the case of Brazil, the effect of private insurance varies depending on care level and income. It is most influential in the middle-income stratum, where individuals with a private health plan are less likely to use primary and emergency care services and more likely to use outpatient secondary care: the PR of those with insurance to those without is 1.57 (CI 95% 1.23-2.00) (Table 5). Having a private health plan implies a greater likelihood of using the three preventive services in all income strata (Table 5).

### What are the effects of income on each health subsystem?

Lastly, the presence of inequities was evaluated according to insurance scheme in Colombia and type of coverage in Brazil (Table 6). In Colombia, the prevalence values for services utilization are consistently higher in the contributory scheme than in the subsidized scheme in all income strata, with the exception of emergency care and caries prevention. In the contributory scheme, level of income does not reveal inequity in health services utilization, except in the cases of outpatient secondary care and breast cancer prevention, which are used more frequently among higher income individuals. In the subsidized scheme, no inequity was detected according to level of income in use of the curative services, but it was observed in the preventive services such as caries prevention (PR comparing those in the middle income stratum with those in the lower is 1.45, CI 95%: 1.12 - 1.87) and breast cancer prevention (PR = 2.00, CI 95%: 1.28 - 3.11) (Table 6). This analysis could not be performed in the uninsured group due to its low level of use of the health services.

In Brazil, higher-income individuals are less likely to use the primary care level whether they hold a private health plan or not. In outpatient secondary care there is an income gradient in individuals with double coverage: the higher the income, the higher the probability of utilization (PR = 1.94, CI 95%: 1.22- 3.10 and PR = 1.98, CI 95%; 1.23 - 3.20) (Table 6). The prevalence of use of preventive services are higher in the group with double coverage (SUS and PHP), and the relationship with income is significant in the case of caries prevention (in both groups), as well as in mammography in the group without private insurance (Table 6).

## Discussion

Twenty years after the reforms implemented to improve equity in access to health care, in both countries inequities persist, in terms of an unequal use for equal need. Although caution should be exercised when drawing generalizations from its results, this study, conducted in two areas in the northeast of Brazil and two in central Colombia, reveals the presence of inequities according to per capita income, possession of a private health plan and SGSSS insurance scheme in the study areas analyzed, where a better socioeconomic status favors utilization of the health services.

In the Colombian areas inequities are found in favor of individuals of a higher socioeconomic status in the three care levels and in the use of preventive services; whereas in the Brazilian areas, the use of secondary services and preventive activities is unequal in favor of the higher-income population, and lower-income individuals make greater use of the primary care services.

In terms of the strengths of the study, we should highlight that this is a comparative analysis based on primary data and with a common questionnaire in which the questions and the recall period are identical, thus avoiding the methodological limitations of international studies based on secondary data which have arisen in other comparative articles [41]. It has therefore allowed us to compare the inequities in access in two countries with different health systems, as well as to fill a current gap in the literature with regard to inequities in the utilization of different care levels and preventive services.

The inequities found would have been greater if the general population had been the object of the study: firstly, due to the fact that the study was also oriented to analyzing barriers in access to the health services of the General System of Social Security in Health (SGSSS) and the Unified Health System (SUS) [62], so areas were selected in which the use of these was predominant over the use of private health services, in other words, areas in which there is a concentration of the low to middle-low income population; and secondly, because the study population was made up of individuals with some kind of health problem or a perceived healthcare need in the three months prior to the interview, and the perception of need for care is greater in the groups with the lowest socioeconomic status than in the general population.

### (In)equity in healthcare services utilization in Colombia

One of the successes attributed to the Colombian reforms is an increase in the population enrolled in the SGSSS, as this was considered to facilitate access and use of the health services [7,27,38]. Although the uninsured population fell from 41.5% in 2000 to 12.1% in 2010 [15], there is a lack of consensus in the literature on the impact this has had on equity in utilization [39]. Our results coincide with those studies in which authors show that enrollment in the SGSSS improves access to the health services [24,27,42], to the extent that uninsured individuals have practically no access. However, this study reveals that inequities also exist between the insurance schemes. Individuals of the subsidized scheme, with equal need, make the least use of primary, secondary and preventive care. This result is in contrast with those of other authors who conclude that enrollment guarantees access [37,38], basing this conclusion on a utilization comparison between individuals of the subsidized scheme and the uninsured, without mentioning the differences between the two schemes. Furthermore these inequities are more pronounced in secondary care services, results which agree with the only study available that differentiates between the two care levels [7].

In emergency care, inequities between the two insurance schemes were not found, but they were found between the insured and uninsured, which appears to indicate that the legal obligation of all public and private entities to offer this type of care to everyone is not fulfilled [14], because it depends on the individual’s insurance status.

It is interesting to note that the role of the insurance scheme is more evident among lower-income individuals, as in these strata it acts as a source of inequity in the utilization of preventive and curative services (with the exception of emergency care), whilst in the higher-income population no differences according to insurance scheme were found in the utilization of services. Moreover, no inequities according to income were found in individuals belonging to the same scheme. In other words, health services utilization by the poorest population – the one which has the greatest need for it – is determined by insurance scheme and not by need.

This study corroborates the argument that the design of the SGSSS displays a structural inequity [1,39], i.e. individuals under the subsidized scheme use the health services less than those under the contributory scheme because the benefits package offered is smaller. Thus the principle of horizontal equity is violated by the design of the health system itself, as it determines that the use of health services depends on the type of SGSSS scheme the individual is enrolled in instead of on the individual’s health needs. It will be necessary to analyze what has occurred since the new law came into effect to make the benefits package equal in the two schemes [63].

### (In)equity in health care services utilization in Brazil

In the case of Brazil, this study is consistent with previous research in terms of the existence of inequities in access to the health services in spite of the fact that it has a national health system [25,26,64], and it also contributes new results: inequity in utilization varies according to care level and is more pronounced in outpatient secondary care and preventive services. There are authors who point out that inequity in access has been reduced in recent years [5,6], although this statement should be treated with caution as they do not distinguish between care levels.

At the primary care level there is inequality in favor of the poorest (i.e. it is pro-poor), meaning that lower-income individuals make greater use of this level, which is the direct opposite of the situation in secondary care. This phenomenon has been described in previous research conducted in other countries with a national health system [8,32,33,65]. Inequity in health care utilization is concentrated at the secondary level, which might be due to the fact that higher-income individuals access private secondary care services directly (via a private health plan or out-of-pocket payment) instead of accessing secondary care through the SUS [66], in order to avoid the significant access barrier of long waiting times in the SUS [62,67]. The lack of specific studies differentiating between these two levels of care in Brazil hinders the comparison of results.

In emergency care no inequities were observed, although the utilization prevalence shows that use falls as per capita income rises and may signal a higher level of use among the poorest, which could be the consequence of the presence of access barriers at the primary care levels, such as the lack of doctors or low levels of health problem resolution reported in other studies [62].

Furthermore, the results show that people with a private health plan have more access to all levels of care than those without, thus the private health plan facilitates access to services [65], but nonetheless income inequalities persist within this group. This may be a result of the existing inequalities in relation to the coverage offered in private health plans, since higher-income individuals pay for more expensive plans, which offer better coverage in terms of complementary tests and medical exams [64].

### (In)equity in preventive activities in both countries

In terms of preventive activities, our study once again reveals inequities in both countries that benefit individuals with a higher socioeconomic status. Only a few studies in Colombia on access to preventive activities analyze specific activities, and those assess inequalities in access to mammography [68] or cervical cytology [69], which hinders their comparison with our results. De Charry et al. pointed out that women in the contributory scheme reported higher levels of mammography services utilization [68]. In Brazil, some studies identify lower odds of having had a mammography among women with a low educational level, low family income and no private health plan [70,71].

Moreover, as our results in Brazil corroborated, inequalities have been described in the preventive use of dental services in the south of the country [72] and other authors reveal inequalities in the use of dental services without differentiating between curative and preventive visits [5]. Furthermore, studies in both countries that do not distinguish between the types of activity identify that the higher the individual’s socioeconomic level, the higher the probability that the medical visit was for preventive rather than curative reasons [7,28,35]. This cycle, in which the poor (lower-income, subsidized scheme and uninsured individuals in Colombia, and those without a private health plan in Brazil) have worse health and worse access to curative health care and preventive activities, makes them particularly vulnerable and may be related to worse health outcomes.

Finally, we should bear in mind that the evaluation of equity in access based on the use of services has been widely criticized for ambiguities in its interpretation: it is difficult to differentiate between necessary use and excessive or insufficient use, both in terms of quantity and quality of care received [73]. In this study, quality of care has not been assessed, although in both countries some results suggest that care quality varies according to socioeconomic level. In Colombia, fewer subsidized scheme enrollees reported that their health problem was solved than contributory scheme enrollees. In Brazil, higher-income individuals reported higher levels of positive outcomes [62]. As there is insufficient data to serve as evidence, further evaluation of equity in access to health services is required, focusing not only on utilization of services but also on quality of care.

## Conclusions

In Colombia and Brazil the health systems have not achieved the equity in utilization prescribed in their laws. Inequity in the utilization of services varies according to care level. It is most noticeable at the secondary care level; only the use of emergency care in Brazil does not show inequities by socioeconomic status. The inequities found in both countries illustrate the close relationship between health services utilization and the design of each health system. Twenty years after the reforms, Colombia, a managed competition model with two insurance schemes, has managed to increase SGSSS enrollment but the benefits packages are still different and remain tied to the purchasing power of the population, which creates conditions of unequal access for equal need. In Brazil, despite universal coverage under the SUS there are still significant inequities in access, especially with regard to outpatient secondary care. The existence of a health system formed by the SUS, steered by principles of equity and universal coverage, together with the private sector, driven by liberalist principles, generates a pattern of inequality in access to the continuum of care. In both countries inequity in the preventive services also points to inequalities at the primary care level.

## Competing interests

The authors declare that they have no competing interests.

## Authors’ contributions

IV and MLV were responsible for the study design and the supervision of all research phases. IG, IV, ASM, MRF and MLV were in charge of the supervision of the fieldwork. IG supervised the data entry, was responsible for the data cleaning, statistical analysis and its interpretation, and drafted the text. IV, CB and MLV contributed to the data analysis and writing of the article. ASM, PdP, MRF, JPU and CB participated in data interpretation. All authors reviewed and approved the final version of the article. The authors alone are responsible for the content of this paper.
